# Guiding principles for the conduct of the Violence Study of Healthcare Workers and System (ViSHWaS): Insights from a global survey

**DOI:** 10.7189/jogh.14.04008

**Published:** 2024-01-12

**Authors:** Tanya Amal, Akshat Banga, Gaurang Bhatt, Umme Habiba Faisal, Aisha Khalid, Mohammed Amir Rais, Nadia Najam, Salim Surani, Faisal A Nawaz, Rahul Kashyap, Global Remote Research Scholars Program

**Affiliations:** 1 Maulana Azad Medical College, New Delhi, India; 2 Global Remote Research Scholars Program, Saint Paul, Minnesota, USA; 3 Sawai Man Singh Medical College, Jaipur, Rajasthan, India; 4 All India Institute of Medical Sciences, Rishikesh, Uttarakhand, India; 5 All India Institute of Medical Sciences, Kalyani, West Bengal, India; 6 Harvard Medical School, Boston, Massachusetts, USA; 7 Faculty of Medicine of Algiers, Algiers, Algeria; 8 Hamdard College of Medicine and Dentistry, Karachi, Sindh, Pakistan; 9 Texas A&M, College Station, Texas, USA; 10 Anesthesiology and Critical Care Medicine, Mayo Clinic, Rochester, Minnesota, USA; 11 Al Amal Psychiatric, Emirates Health Services, Dubai, United Arab Emirates; 12 Department of Research, WellSpan Health, York, Pennsylvania, USA

## Abstract

**Background:**

Although many studies worldwide have reported on violence against health care workers, there is a lack of homogeneous data for understanding the current state of the issue. Conducting a global survey required a robust team organisation structure, unique dissemination strategies, and continual networking to maintain and propagate the pool of survey collaborators and responders. Here we aimed to describe the strategies that helped us carry out a global survey-based study, the lessons learned, and provide a practical roadmap for future large-scale cross-sectional studies.

**Methods:**

We conducted this cross-sectional survey-based study from 6 June to 9 August 2022, basing it on the ‘Hub and Spoke’ model, with a single core team and subgroups in different regions managed by country leads. The key steps included team organisation, strategy formulation for survey dissemination and data collection, social media launch, and conducting a post-survey analysis amongst the collaborators. The core team convened weekly via video conference to discuss the modus operandi. The language barrier was managed through audio translation or by shifting to ‘an interviewer-administered’ questionnaire.

**Results:**

The core team included 11 members from seven countries, followed by 28 country leads from 110 countries. We also gathered 80 regional collaborators who provided feedback and spread the message. The Violence Study of Healthcare Workers and Systems (ViSHWaS) returned 5500 responses globally. Guiding principles garnered through this collaborative project include focusing on effective team organisation, ensuring external validation of survey tool, personalised communication, global networking, timely communication for maintaining momentum, and addressing regional limitations. The post-survey analysis showed that WhatsApp messaging was the most common modality used for survey dissemination, followed by in-person meetings and text messaging. We noted that the successful techniques were direct communication with respondents, regular progress updates, responsiveness to regional and country lead needs, and timely troubleshooting. The most common barriers for the respondents were limitations in language proficiency, technical fallouts, lack of compliance with, and difficulty understanding the questionnaire.

**Conclusions:**

In this global survey-based study of more than 5500 responses from over 110 countries, we noted valuable lessons in team management, survey dissemination, and addressing barriers to collaborative research.

Violence against health care workers (HCWs) has emerged as a significant global challenge, representing a major concern for practitioners, society, organisations, and patients [1]. It can present in many forms, ranging from verbal aggressions to serious physical assaults [2], as well as cyberbullying through using various social media platforms [3]. There is also internal violence committed within hospitals by employees such as moral, racial, or sexual harassment, exacerbated by conflicts between individuals or teams [4]. Importantly, the prevalence of violence against HCWs appears to have increased significantly since the pandemic [5].

While there have been ample records of surveys of violence against health care workers, they are generally focused on a particular country or occupation [3,6]. There is a lack of comprehensive evidence on the spectrum and depth of this problem on a global scale. To address this gap, we formulated a survey-based cross-sectional study targeting HCWs around the globe. The Violence Study of Health Care Workers and Systems (ViSHWaS) is the first of its kind, enrolling HCWs worldwide across various professional hierarchies and systems, aimed at understanding the forms and causes of violence in health care settings [7,8]. Our objective here was to report the project management activities, timelines, and various steps for the implementation of the ViSHWaS. We aimed to discuss the outcomes, factors that aided in successful implementation, and challenges faced, which could act as a guide for the future formulation of mitigation strategies to combat violence in health care settings.

## METHODS

### Study design

This was a cross-sectional survey-based study, designed for thorough recapitulation of all possible genesis of violence among health care workers. The survey contained 23–36 questions which incorporated 12–15 violence, >4 operational, and >8 demographic questionnaires and was prepared using the REDCap tool. Team members who were not part of the survey questions formulation reviewed the questionnaire internally [9]. For instance, the gender question was modified based on internal recommendations to include LGBTQ+ options – ‘Transgender,’ ‘Gender variant/non-conforming,’ and ‘Other/prefer not to disclose’ – to promote gender-inclusive care [10]. After incorporating the given feedback, it was then subjected to external validation [11], which led to changes in the questions evaluating ‘probable causes of violence in health care.’ After successful external validation, the questionnaire was sent to a native-English speaker for evaluation of leading vs non-leading questions. Finally, the survey was launched across the globe on 6 June 2022, and data collection continued until 9 August 2022 (Tables S1–2 in **Online Supplementary Document**). Complete anonymity was ensured for the collected data set. The data was locked in the Excel sheet/REDCap portal accessible only to study investigators.

### Team organisation structure

**Figure 1:**
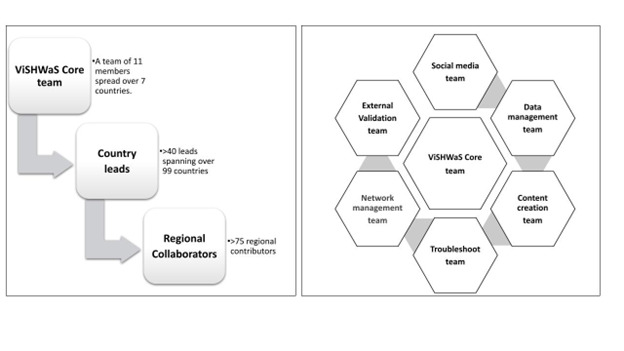
Team organisation structure of core team, country leads, and regional collaborators along with ‘Spoke and Hub’ model.

We required a diverse team representing various nationalities to conduct a global survey like ViSHWaS. Using in-person, instant messaging, e-mail, and video conference exchanges, we assembled a core team comprising professionals from across the medical hierarchy, including doctors, nurses, emergency medical technicians, and medical students **(****Figure 1****)**. For a global-scale study, it was imperative that the core team members be well versed with human subject research regulations; team building and outreach; data collection, data entry, cleaning, and manipulation; epidemiology and data analysis; and statistics [12,13]. The ViSHWaS study used and expanded upon the core competencies in human subject research expertise; team building; and data storage, cleaning, and manipulation that were already present within the Global Remote Research Scholars Program (an existing remote research team) [14]. We organised the members into separate teams based on the ‘Hub and Spoke’ model [15]. The core team convened weekly via video conferences to discuss the modus operandi, including, but not limited to dissemination of the questionnaire, the responsibilities of each team member regarding communicating with HCWs from each country, and strategies for data extraction and analysis.

### Dissemination strategy

**Figure 2:**
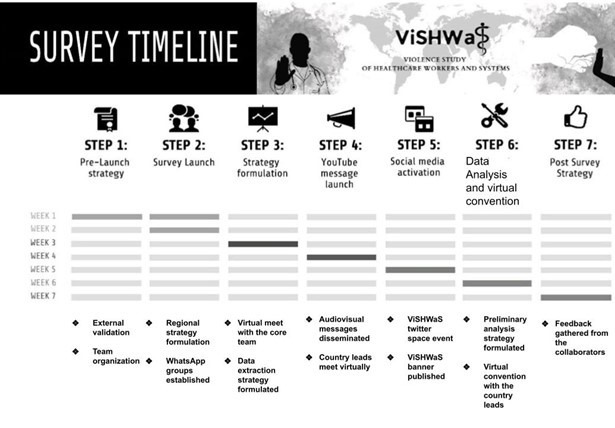
Survey dissemination timeline for ViSHWaS global study.

Initially, a team of 11 people across five countries convened by means of a WhatsApp group, with the aim of disseminating the ViSHWaS globally and acting as the main point of contact for all the contributors and respondents. The momentum of the group was set up by biweekly meetings during which core management strategies were formulated, including promotional ideas, division of responsibilities, and the template for the survey message. A common message was created for the survey which was spread via text, audio, and video messages. The core team comprising the hub (composed of individuals from across the medical hierarchy, including medical students, emergency medicine technicians, attending physicians, etc.) went on to recruit the next layer of country leads via directed messaging **(****Figure 1****)**. Call for leads was also made via YouTube telecasts [16]. Criteria for collaborative authorship were created to attract potential contributors. The country leads contributed by bringing responses from their respective countries and by adding another layer to our team structure by inviting contributors from their and other countries. The banner was created keeping in mind the global nature of our project, which was used as the face of our study in WhatsApp messages, Twitter space events, and LinkedIn posts **(****Figure 2****)**. Our dissemination strategy was vital to the success of our project, as it helped build a strong, global network of collaborators (Table S3 in **Online Supplementary Document**).

### Social media platforms

Communication via WhatsApp alone helped us gather the initial 1000 responses during the first two weeks of our project. We used the ‘snowball technique’ to disseminate our message, with the core team reaching out through approximately a few hundred messages, thus setting the ‘snowball’ effect into action. The country leads were recruited via WhatsApp, LinkedIn, and Twitter, resulting in simultaneous parallel streams of dissemination. The universality of WhatsApp, along with the ability to formulate personalised messages targeting individuals and groups, proved useful in highlighting the importance of our study. Converting the message into regional languages and adding phrases that related to the cohort being addressed (i.e. medical students, physicians, nurses, etc.) and attendings helped in convincing the respondents to contribute to the study. Besides messaging, we also used WhatsApp to create subgroups pertaining to different areas, including survey message template designing, data analysis, and managing country leads.

Apart from WhatsApp, we resorted to other social media platforms. For example, we used YouTube videos to garner attention and motivation for prospective country leads [16] and Twitter as a method to connect with the respondents and discuss various aspects of the survey. Emails and word of mouth also proved useful in attracting collaborators from some countries.

### Addressing potential barriers

The core team members went through the survey along with regional leads during the initial virtual meetings to get them accustomed to it and to answer any of their concerns. This allowed the regional leads to tackle any concern observed by the respondents in an efficient manner; certain problems were identified and addressed during the study. During the process of administering this survey, core team members interacted with individual regional leads on a regular basis to assess and rectify any of the observed concerns. We organised regular virtual meetings via platforms like Zoom, Google Meet, WebEx, Microsoft Teams, and WhatsApp to address any difficulties encountered by the respondents while filling out the survey. For example, owing to the language barrier in many of the francophone and hispanophone regions, we shifted the survey from a self-administered to an interviewer-administered questionnaire. In this way, connecting with respondents on an individual basis helped advance the project. Likewise, taking time to explain the survey questions was crucial for receiving complete responses. We prepared brief translations in French and Spanish explaining the purpose of the survey and disseminated them among the prospective respondents. Midway through our journey, we created audiovisual messages translating the survey and its message to the local language of the respective countries, which we supplemented with personal communication and in-person meetings to ensure that any barriers were overcome.

### Post-survey strategy

**Figure 3:**
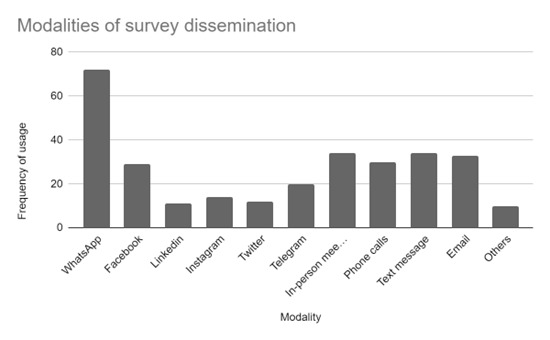
Modalities used by the collaborators according to the post-project survey.

We designed a questionnaire and sent it to all the collaborators to assess their experience in recruiting ViSHWaS survey participants worldwide. Apart from demographic information about the leads, we gathered data on the methods they used to disseminate the message for ViSHWaS. WhatsApp was reported as the most common platform used for disseminating the survey, followed by in-person meetings, text messages, phone calls, LinkedIn, and Instagram **(****Figure 3****)**. The collaborators elaborated on the strategies that seemed to augment the success of the ViSHWaS project. We observed an unanimously positive response to the in-person meeting. Direct messaging and phone calls were the next most favoured strategies. The collaborators felt that contacting the respondents in-person helped them with highlighting the survey’s importance. Adding anecdotes from real-life experiences and accentuating the small amount of time required for the survey served as useful motivational tools. Sending reminders to the potential respondents after the dissemination of messages was important for ensuring the completion of the survey while also allowing the collaborators to address potential barriers to survey dissemination. Highlighting the survey deadline was another method used to ensure compliance.

Networking within health care using the snowball technique was both a tool and a consequence of this survey, with the contributors effectively employing and expanding their network to gather survey responses. This helped them cast a wide net which subsequently led to the incorporation of 110 countries in the survey in a limited period of six weeks. Maintaining the momentum in a ten-week-long project was challenging. The collaborators agreed that, in the short term, the daily updates on WhatsApp helped them to stay motivated, while in the longer term, the weekly virtual meetings with the team helped everyone to stay focused on the goal.

### Ethical approval and previous publications

The Institutional Review Board granted exemption from review to the study proposal. An initial version of the manuscript abstract has been submitted and accepted at The American Thoracic Society International conference in Washington, D.C., in May 2023 [17]. Furthermore, multiple regional subsets of the ViSHWaS manuscript have been submitted and accepted as abstracts for presentation at various national and international conferences [18-20] and in preprints [7,21,22]

## RESULTS

### Survey method outcomes

**Figure 4:**
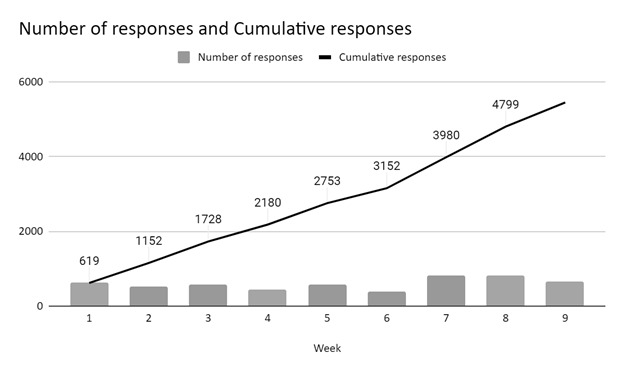
Response timeline of the survey with major milestones.

We collected 5500 responses from 110 countries during eight weeks of the study period. We observed an alternating trend in the weekly survey response timeline, which could possibly reflect the time taken for disseminating the survey, after which each peak was noted. Furthermore, the overall weekly responses increased from approximately 500 responses per week during weeks one to six to approximately 800 per week during weeks six to seven **(****Figure 4****)**. This is in line with the expansion of our global network by recruiting additional collaborators.

### Barriers for respondents

**Figure 5:**
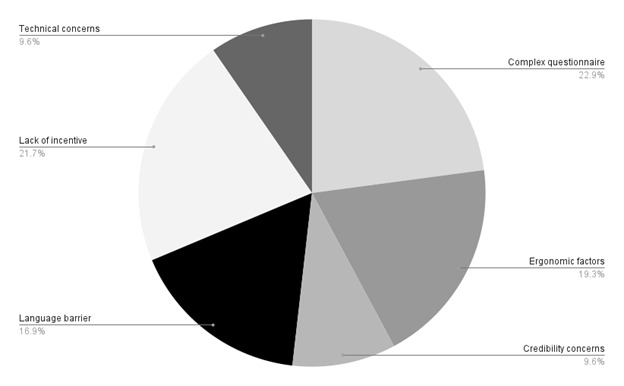
Barriers in recruiting survey respondents based on the post-project survey.

The most common barrier the respondents faced was the difficulty in registering their responses for the question addressing the ‘probable causes’ resulting in violence faced by HCWs, asking for participants to rank the probable cause out of the list provided from Rank 1 (most important in the respondent’s opinion) to Rank 10 (least important in respondent’s opinion) **(****Figure 5****)**. Apart from challenges regarding the technique of assigning a unique number to each cause, the respondents were dissatisfied at not being able to choose multiple causes for the same rank number. This accounted for many of the incomplete responses received by the data collection team. We attempted to attenuate this difficulty by formulating and spreading audiovisual messages explaining the question in detail.

The collaborators felt that the survey lost potential respondents due to a lack of prior trust, particularly if they were approached by someone unknown/less known to them. Despite its anonymity, there was scepticism regarding the legal obligations of responding to the survey. However, this varied depending on the rules and policies of the respective nations. The survey being in English proved a significant barrier in nations where the language was not in common usage. Moreover, the timing clashed with vacation season in some nations, leading to a loss of potential dissemination through the workplace.

One of the barriers observed specifically while disseminating the survey and data collection in resource-limited nations was the lack of access to quality internet, which is essential for an online survey. Complimentary to this were the technical fallouts, with some responses not being registered despite being submitted. Consequently, the form had to be re-administered, leading to impatience among the respondents. The collaborators reported noncompliance with the survey response due to various factors. Lack of incentive was a prominent barrier. Some of the respondents lacked interest and awareness regarding the problem, while others felt uncomfortable due to the sensitivity of the issue. Some responded that the survey questionnaire was long and complex, thus making it less user-friendly. Added to this was the lack of time and recall bias owing to the busy schedule in health care.

## DISCUSSION

### Guiding principles for a global survey study

A global project like ViSHWaS stands on multiple pillars. Throughout the process, we learned from and rectified our mistakes, with each rectification serving as a stepping stone to the next milestone. Here we present some of the guiding principles that should be kept in mind while conducting a global survey.

### Effective team organisation

A robust and active team of leaders is crucial to carrying out the survey [23]; together with the team and through social media [24], we were able to reach out worldwide. The enthusiasm of the core team was reflected in the next layer of leads they recruited. A key to maintaining motivation was connectivity through the WhatsApp group and subgroups [25]; they were instrumental in promoting peer motivation which ultimately kept the survey process active. Daily response updates were seen as rewards and reminders for the collaborators.

### Ensuring external validation

Although we conducted an external validation through a comprehensive team of HCWs [11], we were unable to predict the shortcomings of the ‘ranking questionnaire’ assessing the probable cause of violence. Given the time limitations among health care practicioners, efforts should be made to keep the survey user-friendly. For example, we used the Likert scale in other questions without facing similar problems [26].

### Personalised communication

Direct conversation with potential contributors is the best way of setting up a robust wave of responses through snowball sampling. Besides adding trust to the process, a personal conversation is able to highlight the importance of the problem the survey addresses [27]. Additionally, maintaining direct contact with the respondents is crucial for sending reminders to ensure compliance [28]. Other issues could be related to the timing of sending the questionnaire and the length of the questionnaire also proved challenging, as some respondents were either occupied at work or were unavailable due to vacations [29].

### Global networking

While a direct approach to communication worked effectively for this study and previous studies conducted by the co-authors [30,31], addressing larger groups at a time helped in building a network within health care which would be instrumental for future surveys.

### Timely dissemination

We started disseminating information on the survey about two weeks before its launch. Many of the country’s leads were recruited after the launch of the survey. This was a short period, given the necessity of reaching a global audience. Intimating potential country leads and respondents before the launch of the survey would have created anticipatory excitement and improved outcomes [32].

### Maintaining the momentum

Although the sensitivity of violence in health care was a major factor driving our collaborators, incentives for networking and research opportunities were influential in attracting and maintaining support for the survey [32]. Thus, it is imperative to consider the interests of the contributors, particularly since it is a time-consuming process. Creating incentives kept morale high and drove this survey successfully without the need for funding. The communality convened by the weekly virtual meets has resulted in a consistent and motivated team of collaborators which would be instrumental for future projects [33].

### Limitations

While targeting a global audience, we faced several barriers [34]. Proficiency in the English language was a major limitation of the survey. Thus, given resources and a diverse team, it would be useful to convert the survey questionnaire and the message into the major regional languages [35], which would indicate consideration for the respective population Another notable aspect is the difference in time zones, which created challenges in organising meetings between collaborators and in sharing the survey globally [34].

Inadequacy in internet access was the chief technical limitation experienced in some areas. Alternate techniques like phone calls to conduct an interviewer-administered survey could be a possible alternative [35]. However, this requires assigning a longer time window for survey completion in such regions. Additionally, although there is a potential selection bias risk, as some individuals may be more inclined to participate due to their personal experiences with violence, while others may refrain from participating due to reluctance or ethical concerns, about 30% of survey respondents reported no personal experience with or witnessing workplace violence among colleagues. This aspect of our survey data is significant, as it provides insights from individuals unaffected by workplace violence. Lastly, a unified survey may not be enough to conclude the magnitude of this important issue because of the differences in the definition of violence in different regions.

## CONCLUSIONS

In this global survey-based study of more than 5500 responses from over 110 countries, we gained valuable lessons in team management, survey dissemination, and addressing barriers to collaborative research. The project period was carefully divided between developing a pre-launch strategy, survey dissemination, and assimilation of responses. We therefore recommend incorporating the guiding principles from this study to design future global-scale surveys.

## Additional material


Online Supplementary Document
