# Supplementary material for: Guiding principles for the conduct of the Violence Study of Healthcare Workers and System (ViSHWaS): Insights from a global survey
Source: J Glob Health. 2024 Jan 12;14:04008. doi: 10.7189/jogh.14.04008 (PMC10783207; doi:10.7189/jogh.14.04008)
Supplement: Online Supplementary Document [file jogh-14-04008-s001.pdf]

Online Supplementary Documents

Table S1. Results of Internal Validation of the Survey by 6 Editors

Serial No.	Internal Validator (GRRSP Team member)	Question Modified	Modification
1	Editor 1	1. Divide Questions into Demographics and workplace data<br>2. Increase the options to provide shorter intervals for a better idea	1. Questionnaire split into 2 sub-parts<br>2. Options increased from 3 to 6
2	Editor 2	1. Add: Cultural Violence and Emotional Violence in Type of Violence Questionnaire<br>2. Add: Transgender and Gender Variant in Gender question	1. Cultural violence and emotional violence added as options<br>2. Transgender and Gender Variant/Non-conforming added in Gender question
3	Editor 3	1. Add Online harassment or cyberbullying as an option in the type of violence experienced question<br>2. Add potential question – "Did the abuser have a history of any of the following: Drug abuse; Alcohol Abuse; Psychiatric illness (or hospital admission for psychiatric illness); History of violent acts/manhandling; All; None	1. Cultural violence and emotional violence added as options<br>2. Modified the question and added as a 10-pointer question on the possible cause of violence
4	Editor 4	1. Add a question on the availability of violence reporting procedures<br>2. Add regression analysis using Females and Institution settings as primary predictors	1. Question added<br>2. Regression analysis was performed
5	Editor 5	1. Add a question on the age group of patients dealt with (e.g.: Neonates, children, Adolescents, Adults, Geriatric)<br>2. Add a question regarding the association between night shifts and frequency of violence	1. Suggestion rejected; question not added to keep the survey concise<br>2. A question was added on the association between night shifts and frequency of violence
6	Editor 6	1. Change the following questions to Likert Scale-based questions:<br>A) Worry about violence in the workplace<br>B) Preparedness for a potentially violent situation	1. Likert scale added<br>2. Question modified, and options increased from 2 to 6

		2. Change the question “Violence at the workplace affected me?” to “Effect of violence on perception of career” and change from yes/no, add options.<br>3. Add a question on the department of work	3. A question was added and was part of the survey; however, it was removed from final analysis due non-significant response rate.

Table S2. Results of External Validation of the survey by 10 reviewers

		Workplace Questions														Demographic Questions							
		Q1: Experienced Violence - You or Colleague																					
Serial No.	External Validator (GRRSP Team member name)	1. A) Form of violence	1. B) Number of episodes	1. C) Social Media platform (optional Q)	1.D) Form of Online Harrasment (Optional Q)	1. E) Incidents reported	1. F) Frequency during COVID	1. G) Effect on profession	2. OSH Guidlinese	3. Violence Reporting Procedures	4. Violence management training	5. Worry about workplace violence	6. Prepared for a violent situation	7. Potential causes/risk factors of violence against HCW	8. Night shift work	10. Age	11. Gender	12. Race	13. institution location	14. institution description	15. total years of experience	16. Role at the institution	Additional Comments
1	Reviewer 1	-	-	-	Could ask this question here: "WHAT TYPE OF AGGRESSOR DID YOU ENCOUNTER MOST FREQUENTLY?" Patient, Patient and/or family member, Patient + Relatives and/or carer, Collegue, Supervisors, More than one type of aggressors http://www.clinicaterapeutica.it/2019/170/2/09_CANNAVO.pdf	-	Add option: I don't know or unknown; it could be a choice not all of us know the answer about what happens at our workplace	Add option: I feel less motivated / interested to work / decrease job satisfaction / missing work	-	-	-	-	-	-	-	-	-	-	-	-	-	Add more options - Fellows and medical students	A questionnaire on "Solution for violence against HCW" would be good too. Since there is so much hard work going into this project. We can add this part and get information on suggested mitigation strategies/solutions. This will also become another major part of the survey.
2	Reviewer 2	Replace Emotional Violence with Psychological violence	-	TikTok	-	-	-	-	-	-	-	-	-	Possible questions which can be add here - a. Altered mental state /inebriated state of patient accompaniment. b. Perception that the	-	-	-	-	-	In various countries Millitary service is a must, we can add Millitary hospitals	-	Option - Dentists	-

														assault will be inconsequential for the assailing party<br>c. A general attitude of distrust towards healthcare workers<br><br>Also, it would be helpful if the participants are allowed to allot the same degree of severity to more than one cause.									
3	Reviewer 3	Possible question on the setting of the episode:<br><br>1) Emergency Department<br>2) Inpatient Setting<br>3) Outpatient Department	-	-	-	-	-	-	-	An optional short answer section on how the situation was tackled (e.g.: physical violence - called colleagues /security; emotional abuse - called the hospital's mental health helpline) can be added. It should be	-	-	-	-	-	-	-	Add Middle eastern	-	-	-	-	Is the questionnaire still answearble if we select "NO" in both - violence experienced by self and colleague

										a short answer rather than a multiple choice question as people who have experienced it may be desirous of telling their story.													
4	Reviewer 4	-	-	Add - Linkedin, Telegram	-	-	-	-	-	-	-	-	-	1) Any cause of previous grudge in patients/family members (missed Dx, incorrect Rx, extra charges) 2) Unmet patient needs (already a bullet response but not defined with examples like staff shortages, untrained staff) 3) Financial crisis incurred on patients/family members due to treatment fees,	-	-	-	-	-	-	-	Add - ARNP and physician assistant	-

														sudden loss of job leading to violence as a backlash<br><br>Four Types of Workplace Violence in Healthcare:<br>- Type 1: Criminal Intent<br>- Type 2: Client-on-worker violence<br>- Type 3: Lateral or Worker-on-worker violence<br>- Type 4: Personal Relationship									
5	Reviewer 5	-	-	-	-	-	-	Add options such as moving to a non-clinical career / part-time job	-	-	-	-	-	Clarify the way to fill this. Alternatively, if there is a ranking list where you can drag and drop options that would be simpler to do compared to the current unique ranking of every RF.	Add "seldom" option	-	-	Add: Mixed Race	-	-	-	-	Overall, simple, clear and well structured survey!!!
6	Reviewer 6	-	-	-	-	-	-	-	Option to enter their	Respondents can add a comment	-	-	-	Add additional point - Lack of	-	-	-	-	-	-	-	-	1) Take into consideration local/regional policies regarding

									Regional/country-specific guidelines	about what methods are available in they select "yes"				respect towards healthcare worker									Violent acts against HCWs.<br><br>2) We can give incentives for filling survey
7	Reviewer 7	-	-	-	-	Add hospital staff as the perpetrator	-	-	-	-	-	-	-	Giving the same score to another cause of violence removes the response from the first selected option. Can we allow allotment of the same rank to more than 1 option to resolve this issue?	-	-	Add more options - Homosexual, Bisexual, LGBTQ+, or Non-binary	-	-	-	-	-	Make a separate instruction presentation/video other than the instruction from the tool itself.<br><br>It is a bit long still and could be made shorter
8	Reviewer 8	-	The violent episode frequency is limited to the last 1 year. Can it be more than 1 year	-	-	-	-	-	-	-	Strategies for primary prevention of violence/violent episode	-	-	-	-	-	-	-	-	Add Mission/Non-profit hospitals	-	Add - Researchers<br><br>Add work as full time or part time or internship or volunteer	1) Long and complex questions - Can make the questionnaire shorter.<br><br>2) We can use Google survey instead of REDCap survey

9	Reviewer 9	-	-	-	-	-	-	Can Death from violence be option?	-	-	-	-	-	Add an 11th option as a fill-in-the-blank where the respondent can add their option	-	-	-	Please add: American Indian, Native Alaskan, Pacific Islander	-	-	-	More options - Physical therapist occupational therapist, EMT	English is not the primary language in our country, translation into French would be better.
10	Reviewer 10	Use the term Racial harassment instead of cultural violence	-	-	-	-	Add an option of N/A or Inderminate	-	-	-	-	-	-	-	Can add 24 hr shifts and >24 hr shifts as options	-	-	-	-	-	-	Add Junior resident as an option as a lot of people work as interns and JRs before residency	Spreading the survey through medical societies and forums would improve response rate

Table S3. Country-Wise Statistics of Collaborators and Survey Dissemination Strategies

Country Name	Count (N = 5405)	# Contributors	Method of survey dissemination
Afghanistan	100	2	Emails, Facebook, WhatsApp, Telegram
Algeria	127	1	N/A
Argentina	120	1	N/A
Australia	108	1	Emails, Instragram, WhatsApp, Text messages, In-person meetings, LinkedIn, Twitter
Bahrain	56	3	Facebook, WhatsApp, Text messages, In-person meetings, Telegram, Twitter
Bangladesh	76	4	Emails, Facebook, WhatsApp, Phone calls, Text messages, In-person meetings, Telegram, Other online forums
Bolivia	24	2	N/A
Bosnia and Herzegovina	17	3	N/A
Brazil	49	2	N/A
Burundi	51	2	N/A
Canada	14	2	N/A
China	27	3	Emails, Instragram, WhatsApp, LinkedIn, Wechat
Colombia	40	2	N/A
Croatia	40	2	Emails, WhatsApp
Cuba	26	2	Emails, WhatsApp
Cyprus	101	2	N/A
Democratic republic of Congo	119	3	WhatsApp
Dominica	61	2	N/A
Dominican Republic	31	2	WhatsApp, Text messages
Ecuador	118	2	WhatsApp, Phone calls, In-person meetings
Egypt	76	2	N/A
El Salvador	27	2	Facebook, WhatsApp
Georgia	26	1	N/A
Germany	25	1	N/A
Greece	50	4	Emails, Facebook, Instragram, WhatsApp, Phone calls, In-person meetings, LinkedIn, Other online forums, Twitter
Grenada	31	1	N/A
Guatemala	106	2	N/A
Guyana	53	2	WhatsApp
Haiti	50	2	Emails, WhatsApp, Phone calls, Text messages

Honduras	78	1	N/A
Hungary	11	2	N/A
India	656	12	Emails, Facebook, Instragram, WhatsApp, Phone calls, Text messages, In-person meetings, LinkedIn, Telegram, Other online forums, Twitter
Indonesia	69	1	Facebook, Instragram, WhatsApp, Text messages, In-person meetings, Telegram, Other online forums
Iran	22	3	N/A
Iraq	25	1	N/A
Jamaica	12	2	N/A
Japan	11	1	N/A
Jordan	75	2	N/A
Kenya	44	1	N/A
Kuwait	59	2	Instragram, WhatsApp
Kyrgyzstan	13	1	N/A
Lebanon	17	1	N/A
Libya	54	1	Emails, Facebook, WhatsApp, Phone calls, Text messages, In-person meetings, Telegram
Malaysia	13	1	N/A
Mexico	49	1	N/A
Morocco	65	1	N/A
Nepal	102	3	Emails, WhatsApp, In-person meetings, LinkedIn, Twitter
New Zealand	23	1	N/A
Nigeria	104	4	Facebook, WhatsApp, In-person meetings, LinkedIn, Telegram
Pakistan	154	6	Emails, Instragram, WhatsApp, Text messages, In-person meetings, LinkedIn, Twitter
Palestine	51	2	N/A
Peru	106	2	WhatsApp, Phone calls, In-person meetings
Philippines	61	2	N/A
Poland	32	3	Emails, Facebook, In-person meetings, Twitter
Qatar	52	2	Emails, Facebook, Instragram, WhatsApp, Text messages, LinkedIn, Twitter
Russia	18	2	N/A
Rwanda	35	2	Emails, WhatsApp, Phone calls, Text messages, In-person meetings
Saudi Arabia	88	1	WhatsApp, Phone calls, Text messages
Serbia	33	3	Emails, Phone calls, Text messages, In-person meetings, LinkedIn, Other online forums, Viber group

Singapore	55	2	Instragram, WhatsApp, In-person meetings, Telegram
Somalia	56	2	N/A
South Africa	35	2	WhatsApp, Phone calls, In-person meetings
South Korea	78	1	Facebook, WhatsApp, Text messages, LinkedIn, Other online forums
Sri Lanka	14	1	N/A
Sudan	67	1	WhatsApp, Phone calls, In-person meetings
Switzerland	12	1	N/A
Syria	59	3	Emails, Facebook, WhatsApp, Phone calls, Text messages, In-person meetings, Telegram
Tanzania	56	2	WhatsApp
Thailand	12	1	N/A
Trinidad and Tobago	14	2	N/A
Tunisia	52	1	N/A
Turkey	50	2	WhatsApp, Phone calls, Text messages, In-person meetings, Telegram
UAE	54	1	Instragram, WhatsApp, Twitter
Uganda	103	2	N/A
UK	19	3	WhatsApp, In-person meetings
Ukraine	13	1	N/A
USA	395	12	N/A
Venezuela	168	2	Instragram, WhatsApp, Text messages
Yemen	54	2	Facebook, WhatsApp, In-person meetings, Telegram
